# A high-intensity low-frequency acoustic generator based on the Helmholtz resonator and airflow modulator

**DOI:** 10.1371/journal.pone.0300832

**Published:** 2024-03-22

**Authors:** Baoguo Zhang, Mingrong Dong, Bin He, Houlin Fang, Haozhong Ruan, Min Zhang, Xubin Liang, Fang Zhang, Deyu Sun

**Affiliations:** 1 Northwest Institute of Nuclear Technology, Xi’an, China; 2 Faculty of Information Engineering and Automation, Kunming University of Science and Technology, Kunming, China; COMSATS University Islamabad, PAKISTAN

## Abstract

The high-intensity low-frequency acoustic sources have essential applications in acoustic biological effects research, airport bird repelling, and boiler ash removal. However, generating high-intensity low-frequency acoustic waves in open space is difficult. In this paper, a low-frequency acoustic generator with a resonant cavity used to enhance the acoustic intensity in open space was developed, which is an aerodynamic acoustic generator to radiates a high-intensity acoustic wave of 52Hz. Some experiments were carried out to measure this generator’s internal flow field and radiated acoustic field characteristics, including the propagation characteristics at 100m. The experimental results show that the resonant enhancement effect is presented near the predetermined resonance frequency, and the enhanced value is about 4dB. The acoustic intensity for 52Hz at 1m position is 124dB. By combining the Helmholtz resonator with the airflow modulator, the airflow resonance in the resonator enhances the air pressure pulsation inside the chamber and increases the disturbance of acoustic radiation to the air. So as to improve the sound intensity and radiation efficiency in the low-frequency range.

## Introduction

In recent years, with the gradual expansion of the area of wind power generation, with the increasingly frequent use of high-speed rail, aircraft, and other means of transportation, noise, especially high-intensity low-frequency noise pollution, is becoming more and more serious. These facts promote the research of noise reduction technology and the biological effects of the high-intensity low-frequency acoustic source [1–4]. These studies require artificially controllable high-intensity low-frequency acoustic sources as acoustic sources. In addition, the high-intensity low-frequency acoustic source can be used for boiler ash removal [5], airport bird repelling [6], etc. Although many brands and performance speakers have been used in production and life, it is mainly to achieve medium and high-frequency acoustic wave generation. There are few high-intensity low-frequency acoustic sources in open space because generating high-intensity low-frequency acoustic waves is challenging. Although the speaker can improve the acoustic radiation efficiency by increasing the input electric power, low-frequency acoustic radiation requires a large-size diaphragm and a large-displacement reciprocating motion of the diaphragm, which is difficult to achieve in engineering. Wind farm noise is a low-frequency noise generated by the rotation of a large fan blade, whose intensity is very low and difficult to detect by humans [7]. Therefore, exploring other methods to make high-intensity acoustic sources is necessary.

The aerodynamic acoustic source [8], usually used to make the high-intensity acoustic source, has high radiation efficiency and intensity. The aerodynamic acoustic source, which can reach the acoustic power of ten thousand watts, is usually composed of an airflow modulator and a horn. The principle of the aerodynamic acoustic source is similar to the process of human speech, and the required acoustic wave is generated by modulating the air [9]. In the 1960s and 1970s, Meyer [8] studied the acoustic principle of modulators. With the development of fluid mechanics, computational mechanics, and computer science, the theoretical simulation of the acoustic generation process of the aerodynamic acoustic source can be carried out [10–15]. Due to the complexity of the acoustic wave generated by the fluid, the theoretical simulation may not apply to various working conditions that generate acoustic waves. Therefore, experimental research is also essential for aerodynamic acoustic source research [16, 17]. In addition to the acoustic wave generated by the airflow modulation, which is similar to the human voice, the aerodynamic acoustic source also has the acoustic of jets [18–20], high-pressure air flowing through some components [21, 22], and so on. The acoustic intensity of these aerodynamic acoustic sources can be enormous, but they are mainly high-frequency components. It is challenging to improve the intensity of low-frequency components.

Helmholtz resonator is one of the most basic acoustic resonance systems [23]. The Helmholtz resonators are commonly used in the noise control field for noise reduction [24] and acoustic absorption [25]. Some research involved the nozzle shape of the resonant tubes and the acoustic characteristics inside the resonant tube [26]. The aperture-embedded Helmholtz resonance wall structure’s acoustic absorption performance was greatly enhanced compared with the conventional wall structure [27]. Wu and Guan [28] studied the maximizing method of the resonator’s noise-damping performances by applying and optimizing an extended neck. Gorain and Padmanabhan [29] propose a new broadband acoustic metamaterial (AMM) absorber to broaden the frequency band of the low-frequency noise attenuation of the Helmholtz resonators. Elkhateeb and Eldakdoky [30] studied the acoustic absorption performance of empty plastic bottles. The results show that a remarkably higher level of absorption is achieved in the mid- and high-frequency ranges by randomly placing the 1500 ml bottles onto the room floor. Using the Helmholtz resonator technology, Kamiyama [31] designed a twin-chamber plastic resonator to reduce tire cavity noise. Vigran and Haugen [32] investigate the transmission loss of some prototype circular-shaped silencers and illustrate a method to design the perforated duct walls to optimize the transmission loss in a given frequency range.

On the other hand, among the research associated with the high-intensity low-frequency acoustic generator, using the resonator can improve the intensity of the low-frequency acoustic in a closed space. Boesch, Reiff [33] constructed a high-intensity infrasound generation device using an airflow modulator and a Helmholtz resonator, whose acoustic intensity is greater than 140 dB. Then they constructed a double-chamber resonator system to achieve an acoustic intensity greater than 155 dB [33]. The author of this paper, Dong, Ke-An [34] investigated the working mechanisms of the infrasonic acoustic generator consisting of a modulator and a Helmholtz resonator and manufactured a prototype device with an infrasound pressure level over 162 dB below 3 Hz.

In this study, a modulator with the same round holes in the stator and rotor was designed. The change in the air supply flow rate and pressure were analyzed. An aero-acoustic device was designed using the resonator to enhance the intensity of the acoustic radiation in the open space to improve the low-frequency intensity of the acoustic radiation. Concretely, a low-frequency acoustic generator was designed with the target of 52 Hz acoustic radiation based on the Helmholtz resonant structure, and some experimental research was carried out.

## Device design

### The design principle of the airflow modulator with a turning round throat

**Fig 1:**
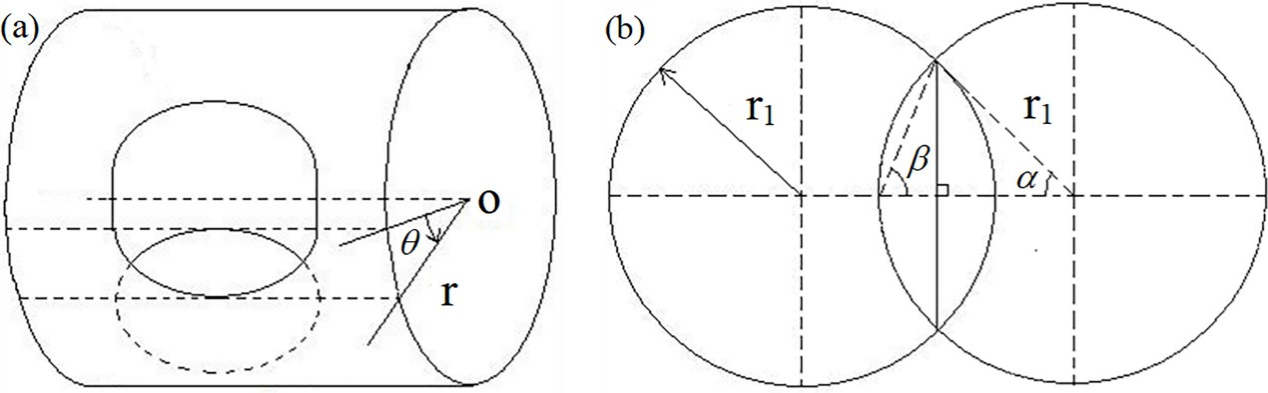
(a) Sketch map of the modulator, (b) Sketch map of the exhaust area intersection.

The structure of the airflow modulator with a turning round throat is simple and easy to realize the small-diameter airflow modulation. Such a modulator has the same holes on the rotor and stator. Let the radius of these holes be *r*_1_. Fig 1a shows the sketch map of the modulator in which the stator cylinder is inside, and the rotor cylinder is outside. Fig 1b shows the sketch map of the exhaust area intersection. The rotor is turned around the cylinder axis of the stator. Let the cylinder radius of the stator and the rotor be *r*. The pipeline connects the inner cylinder with the air chamber, and the servo motor turns the outer cylinder with a specific frequency. When the hole in the outer cylinder intersects with the hole in the inner cylinder, the exhaust throats, which can generate the pulsing jets, are formed and generate the acoustic wave with a specific frequency. The fundamental frequency of this acoustic wave is also called the working frequency. Suppose the same area of the exhaust throats can be divided into several exhaust throats uniformly distributed on the circumference. In that case, the machining is convenient, and the working stability of the modulator can be improved. Let the number of exhaust throats group be *n*.

The throats begin to open when *θ* = 0 and its area can reach a maximum value of $\pi r_{1}^{2}$ when $\theta =2\theta_{r_{1}}$. The $\theta_{r_{1}}$ is given by

$$\theta_{r_{1}}=\text{arcsin}\left(\frac{r_{1}}{r}\right)$$ (1)

Then the area of the throat in one period can be derived as

$$A\left(\theta \right)=\left\{\begin{array}{cc} 2r_{1}^{2}\left[\alpha_{1}-\left(1-\frac{r}{r_{1}}\;\text{sin}\left(\frac{\theta }{2}\right)\right)\text{sin}\left(\alpha_{1}\right)\right] & 0\leq \theta \leq 2\theta_{r_{1}}\\ 2r_{1}^{2}\left[\alpha_{2}-\frac{r}{r_{1}}\;\text{sin}\left(\frac{\theta -\theta_{d}}{2}\right)\text{sin}\left(\alpha_{2}\right)\right] & 2\theta_{r_{1}}<\theta <4\theta_{r_{1}}\\ 0 & 4\theta_{r_{1}}\leq \theta <\theta_{T} \end{array}\right.$$ (2)

$$\alpha_{1}=\text{arccos}\left(1-\frac{r}{r_{1}}\;\text{sin}\left(\frac{\theta }{2}\right)\right)$$ (3)

$$\alpha_{2}=\text{arccos}\left(\frac{r}{r_{1}}\text{sin}\left(\frac{\theta -2\theta_{r_{1}}}{2}\right)\right)$$ (4)

where *θ* = 2*πft* and *f* is the working frequency of the modulator. If *A*(*θ*) is changed into *A*(*t*), the calculation will become convenient.

The mass airflow rate *m*_*a*_(*t*) can be deduced by the thermodynamic fundamental equations.

$$m_{a}\left(t\right)=P_{in}MA\left(t\right){\left[1+\frac{\left(\gamma -1\right)M^{2}}{2}\right]}^{-\frac{\gamma +1}{2\left(\gamma -1\right)}}\sqrt{\frac{\gamma }{RT_{in}}}$$ (5)

where *P*_*in*_ is the pressure in the air chamber, *M* is the liquid Mach number, *T*_*in*_ is the temperature in the air chamber, *γ* is the air-specific heat, *θ*_*T*_ = 2*π*/*n* is the angle range of one period, and *n* is the number of the throats.

The mass airflow rate *m*_*a*_(*t*) can be changed into volume airflow rate as follows

Ftm=matρatm,ρatm=ρ0PatmT0P0Tatm (6)

where *P*_*atm*_ is the environmental pressure, *T*_*atm*_ is the environmental temperature, *P*_0_, *T*_0_ and *ρ*_0_ are the standard condition’s pressure, temperature, and density.

The fluid mechanics theory can deduce the pressure of the air chamber changes with time.

$$P_{t}\left(i\right)=\left\{{\left(\frac{F_{in}\Delta t-F_{m}\Delta t+V_{t}}{V_{t}}\right)}^{\gamma }\left[P_{t}\left(i-1\right)+P_{atm}\right]\right\}-P_{atm}$$ (7)

where *V*_*t*_ is the air chamber volume of the modulator, Δ*t* is the time step, $F_{in}=\frac{F_{c}\left[P_{ma}-P_{t}\left(i-1\right)\right]}{P_{ma}}$ is the input airflow rate of the throats, *F*_*c*_ is the input airflow rate of the air chamber, and *P*_*ma*_ is the input pressure of the air chamber.

If the modulator works at a single frequency, the impedance is changed cyclically following the working frequency, and the impedance is also changed in one period. According to the modulator impedance definition, to find the maximum airflow rate value in one period and then the corresponding value of the pressure, the impedance of the modulator can be obtained by the pressure divided by the airflow rate. Therefore, the impedance considered for the impedance matching is the modulator impedance when the airflow rate is at maximum. Modulator impedance is

$$R_{mn}=\frac{P_{mm}}{F_{mm}}$$ (8)

where *F*_*mm*_ is the maximum airflow rate in one period, m^3^/s, *P*_*mm*_ is the pressure of the air chamber corresponded with *F*_*mm*_.

The airflow rate for the working frequency *F*_*f*_ can be obtained by analyzing the throat’s airflow rate curve *F*_*m*_(*t*). Then, the sound pressure for the working frequency can be given

$$p_{f}=R_{mm}\cdot F_{f}$$ (9)

The sound pressure calculated by the above formulas for the working frequency leads to the fact that the cross-sectional area of the throats is equal to the maximum area of the nozzle. Suppose the cross-sectional area of the throats isn’t equal to the maximum area of the nozzle. In that case, the theory for the acoustic wave propagation in the pipes should be adapted to calculate this sound pressure on the base of the formula (9).

### The characteristic calculation for the throat acoustic wave

To calculate the characteristic of the throat acoustic wave, the basic design parameters for an airflow modulator with a turning round throat are designed as follows: the cylinder radius is 0.05m, and the hole’s radius for the stator is 0.03m. The cross-sectional area of the throats is equal to the maximum area of the nozzle. So the radius of the throat is also 0.03m. The volume of the air chamber for this modulator is 0.1m_3_. The air supply pressure is 328kPa, and the airflow rate is 0.5m_3_/s. The working frequency is 8Hz.

**Fig 2:**
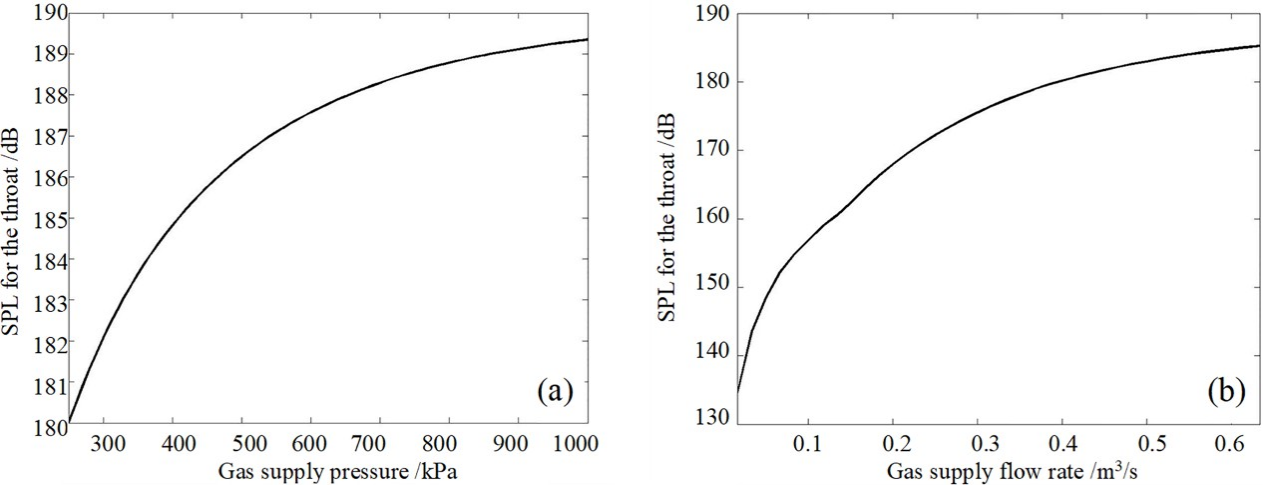
(a) Throat SPL changes with the air supply pressure, (b) Throat SPL changes with the air supply flow rate.

Throat SPL change with the air supply pressure and the air supply flow rate is calculated in Fig 2a and 2b. it is shown that

1. The change in the air supply pressure. The results for the air supply pressure in the range 250kPa-1000kPa show that the throat SPL is increased with the pressure increase from 180 dB to 189 dB. However the increased rate of the SPL decreases with the rise of the pressure, and the curve tends to flat.
2. The change in the airflow rate. The air supply flow rate results in the range 0.01m^3^/ s~0.62m^3^/s show that the larger the flow rate value, the larger the throat SPL value. The throat SPL is 135 dB when the flow rate is 0.01 m^3^/s, and the throat SPL is 185 dB when the flow rate is 0.62 m^3^/s. However, the increased rate of the SPL decreases with the increase of the flow rate, and the curve tends to be flat.

Obviously, the higher the throat SPL, the higher the acoustic intensity radiated in the air. Therefore, improving the acoustic intensity by increasing the air supply pressure and flow rate is helpful. In the working process of the modulator, increasing the pressure in the air chamber and the airflow effect increases the air chamber pressure pulsation. Therefore, the modulator and the resonant chamber are combined to form a resonator. A pipeline is connected with the export of the modulator as a resonant tube. The resonant chamber is equivalent to the air chamber of the modulator. It can generate larger pressure pulsation in the air chamber at the resonant frequency, which should improve the radiated acoustic intensity in theory.

### The design of the airflow modulator with a turning round throat

**Fig 3:**
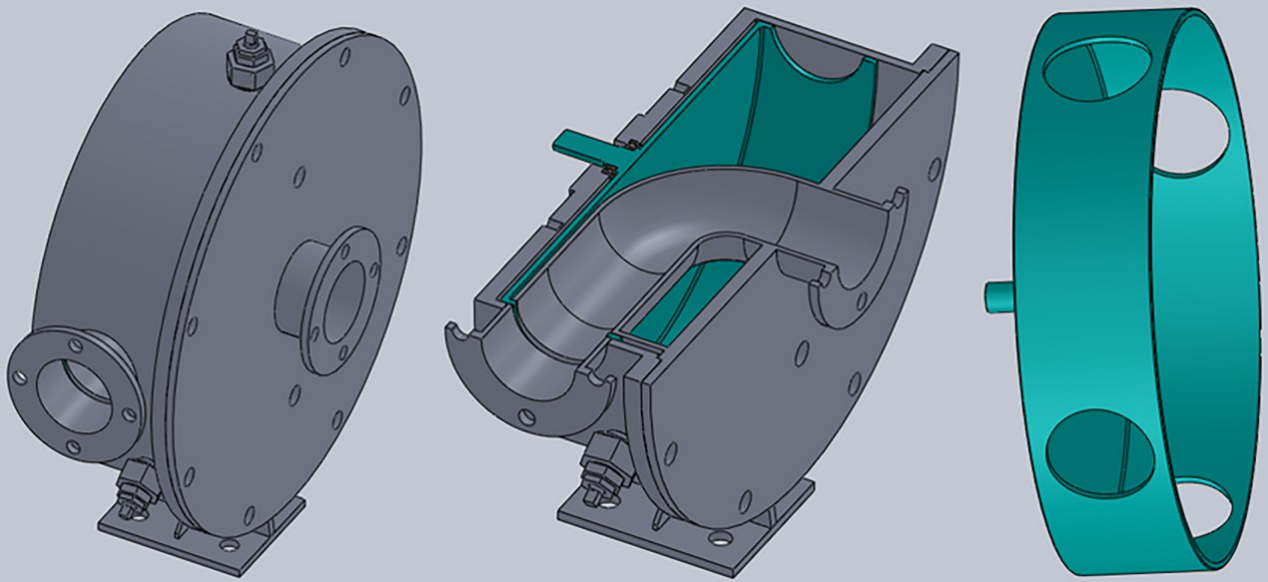
Sketch map of the airflow modulator with turning round throat.

**Fig 4:**
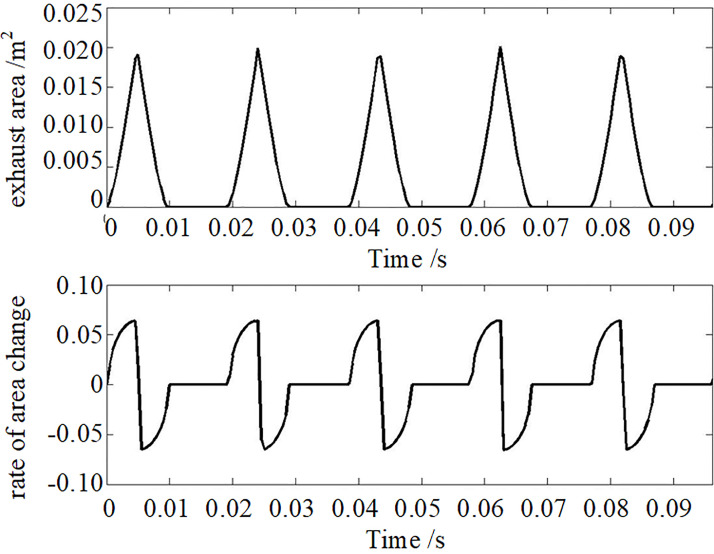
The modulator exhaust area and its rate of change at 52 Hz.

The airflow modulator with a turning round throat can quickly realize the airflow modulation on the pipeline. This modulator is installed on the resonance tube, whose diameter is 0.080 m of the resonator. The modulator is designed as shown in Fig 3. The cylinder radius of the stator and the rotor is 0.205 m. The throat of this modulator is a round hole whose diameter is 0.08m, and there are 4 round holes on the rotor. The rotor is droved by a servo motor to realize the periodic on-off for the airflow in the resonance tube. Fig 4 shows the theoretical calculation results of the modulator exhaust area and its rate of change. Table 1 is the theoretical calculation results of this modulator performance at the working frequency of 52 Hz.

**Table 1:** The theoretical results for the modulator at 52 Hz.

Content	Value
The volume of the air chamber /m^3^	0.0096
The air supply pressure /kPa	240
The air supply flow rate /m^3^/s	0.583
The impedance of the modulator /Ω	7402
The SPL in the air chamber /dB	184
The SPL in the throat /dB	164
The impedance in the throat /Ω	1440
The sound power /W	140

### The structure design and realization for the resonant acoustic generator

**Fig 5:**
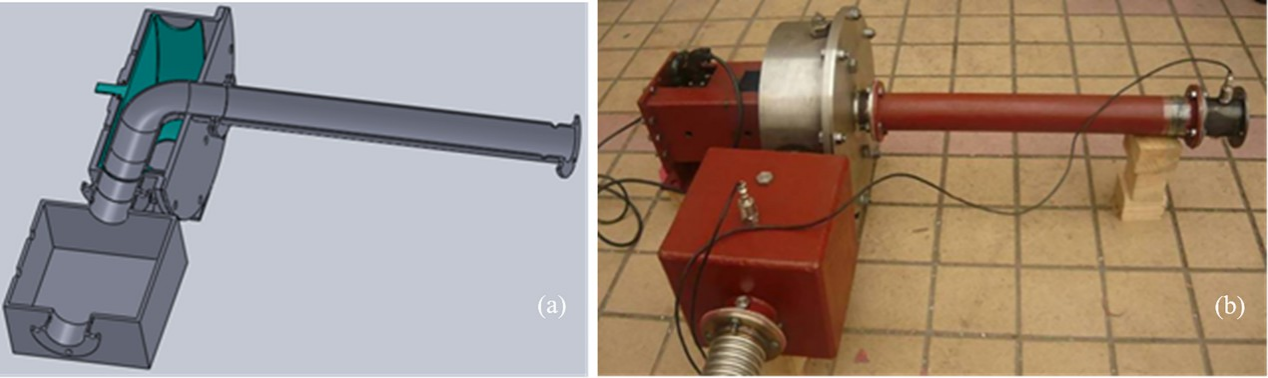
(a) The design sketch map for the resonant acoustic generator, (b) the actual photo of the resonant acoustic generator.

Let’s discuss the structure design parameters of the Helmholtz resonator. The resonant tube’s diameter and length are 0.80 m and 0.56 m, and the volume of the resonant cube chamber is 0.0096 m^3^. The resonant frequency of this resonator is 52 Hz. The throat-cutting airflow is as close as possible to the resonant chamber. The length inside the modulator should be contained in the length of the resonant tube. For convenience of description, this acoustic generator is called a resonant acoustic generator. Fig 5a shows the design sketch map for this device. It can be seen that the inner wall of the resonant tube is still smooth while the modulator is increased near the throat. Fig 5b shows the actual photo of this device. Some thick steel plate welds the resonant chamber. The air inlet pipe and the modulator are connected with the resonant chamber through the flanges.

## Experiment

### A brief introduction to the experimental process

The air source is an air compressor with a flow rate of 35 m^3^/min and an air supply pressure of 240 kPa in the experimental process.

The sweep measurement technology is used in this experiment with a frequency range of 10~69 Hz. One pressure sensor is used to measure the pressure of the air chamber, and the other pressure sensor, mounted in the position 0.050 m distance to the nozzle of the resonant tube, is used to measure the pressure inside the resonant tube. The key of this experiment is to measure the radiated acoustic characteristics in the environment. The acoustic signal in the acoustic beam central axis is measured by B&K microphone type 4193 at 1 m (45 degrees direction), 10 m, 50 m, and 100 m. The NI USB 6210 data acquisition card is adopted, the input voltage range is ±10V, and the sampling rate is 10kHz during the experiments.

### The experimental results

#### The internal flow field and acoustic field characteristics of this device

**Fig 6:**
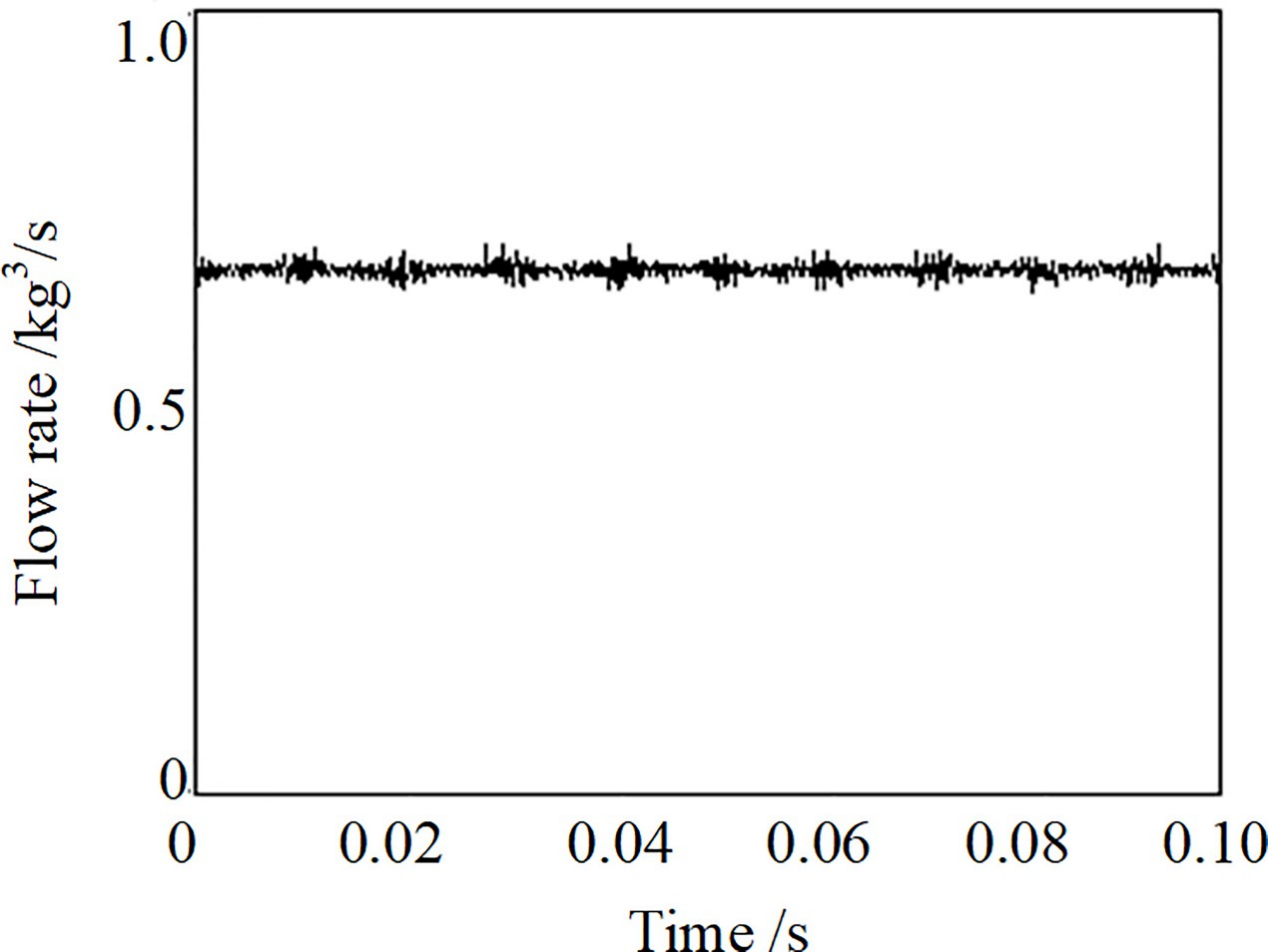
The instantaneous flow rate of the resonant acoustic generator at 52 Hz.

Fig 6 shows the instantaneous flow rate of the working frequency 52 Hz of the resonant acoustic generator. The value of 0.674 kg/s of flow rate is essentially unchanged. There are visible burrs of 52 Hz on the flow rate curve. This phenomenon shows that strong pressure pulsation within the resonant chamber spread to the flowmeter during the experiment process for the resonant system.

**Fig 7:**
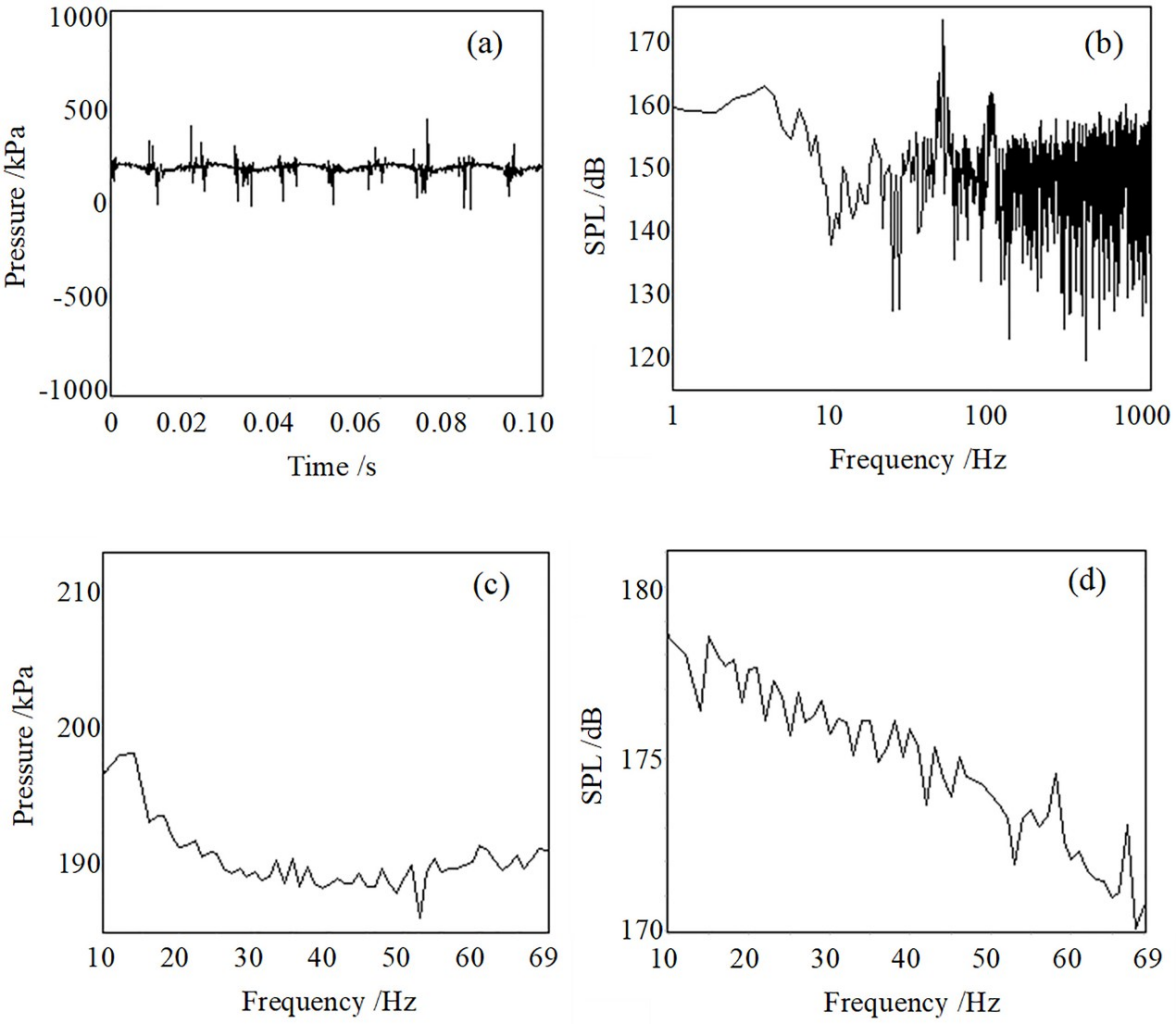
The pressure signal (a) and the spectrum (b) of the air chamber of the resonant acoustic generator at 52 Hz, the static pressure (c), and the spectrum (d) of the air chamber of the resonant acoustic generator.

Fig 7a and 7b show the air chamber’s signal and spectrum with a working frequency of 52 Hz. The SPL of 52 Hz is 173 dB, and the SPL of its second harmonic is 162 dB. Fig 7c and 7d show the sweep measurement results of the air chamber pressure, which means static pressure is about 190 kPa. The static pressure curve has a clear trough of about 186 kPa near the resonant frequency of 52 Hz. The amplitude of the static pressure has a specific degree increase after this trough. There is a trough near the corresponding position in the fundamental SPL curve of the dynamic pressure. A crest on the fundamental frequency SPL curve appeared after this trough. Obviously, the higher the static pressure, the higher the SPL generated by the dynamic pressure. The repeated experimental results all have these similar phenomena, which indicate that the appearance of the trough is related to the system resonance and can be used as one foundation to judge the system resonance frequency.

**Fig 8:**
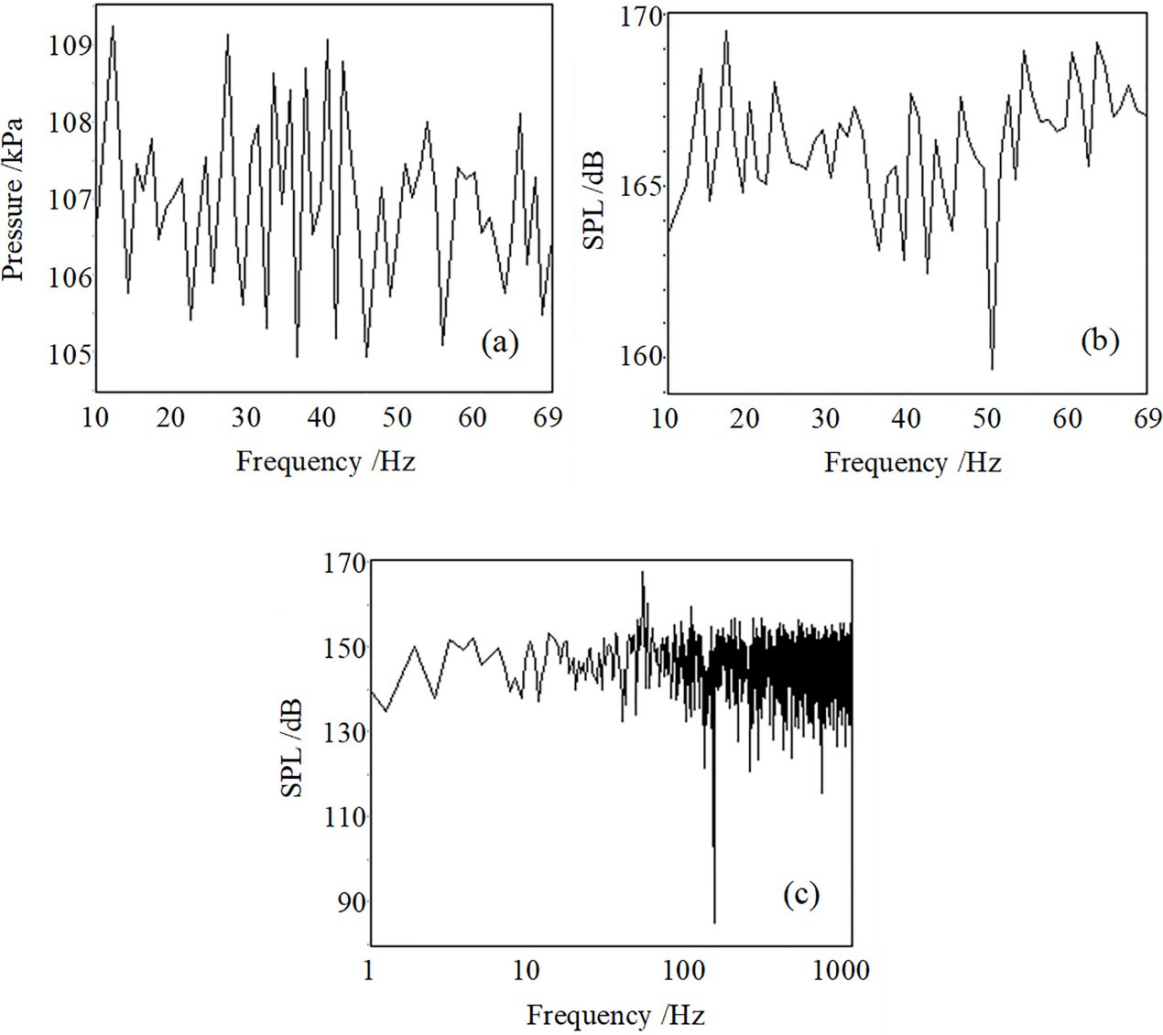
The air static pressure (a) in the nozzle of the resonant acoustic generator’s resonant tube and its spectrum (b), the spectrum of the air pressure signal in the nozzle of the resonant tube at 52 Hz (c).

Fig 8a and 8b show the sweep measurement results of the air pressure in the nozzle of the resonant tube. It means that static pressure is about 107 kPa and is approximate to ambient pressure (1 atm). The static pressure curve has fluctuations of about 1 or 2 kPa, which belong to regular changes and show the measurement error of the pressure sensor. The curve of the fundamental frequency SPL of the dynamic pressure has similar fluctuations with an amplitude of about 2 dB. Fig 8c shows the spectrum of the air pressure signal in the nozzle of the resonant tube’s working frequency of 52 Hz. The SPL of 52 Hz is 168 dB, and the SPL of its second harmonic is 155 dB.

#### The measurement results of the acoustic at 1m

**Fig 9:**
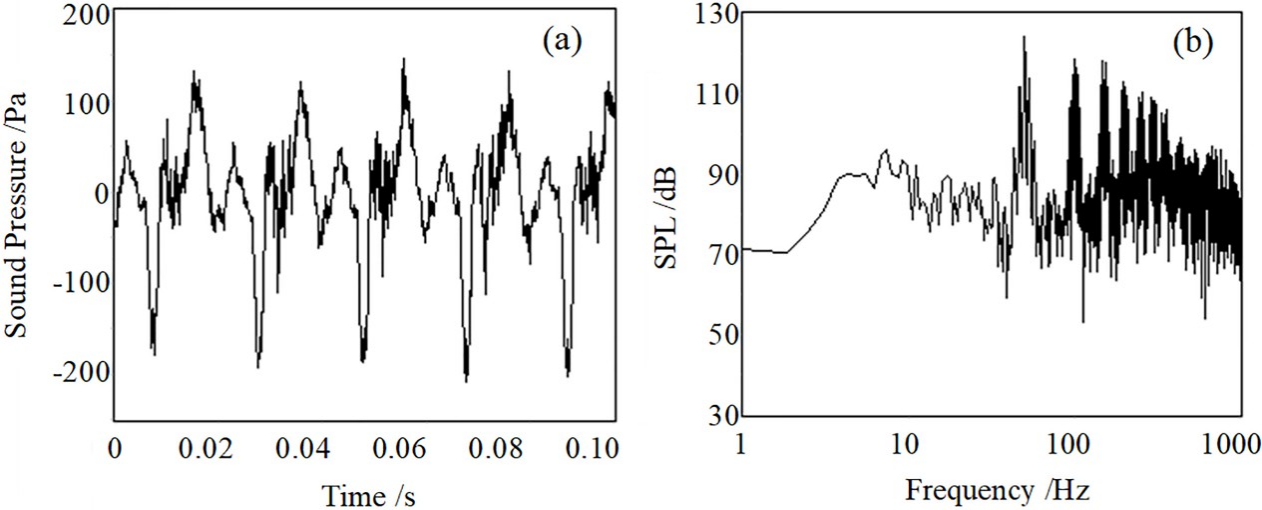
The acoustic signal (a) and the spectrum (b) at 52 Hz at 1m (45 degrees direction).

Fig 9a and 9b show the typical acoustic signal and spectrum of the resonant acoustic generator of the working frequency 52 Hz at 1 m (45 degrees direction). The SPL of 52 Hz is 125 dB, and the SPL of its second harmonic is 117 dB. There are apparent pressure pulses formed by the jet in the signal. The acoustic intensities for frequencies above 100 Hz are about 95 dB.

**Fig 10:**
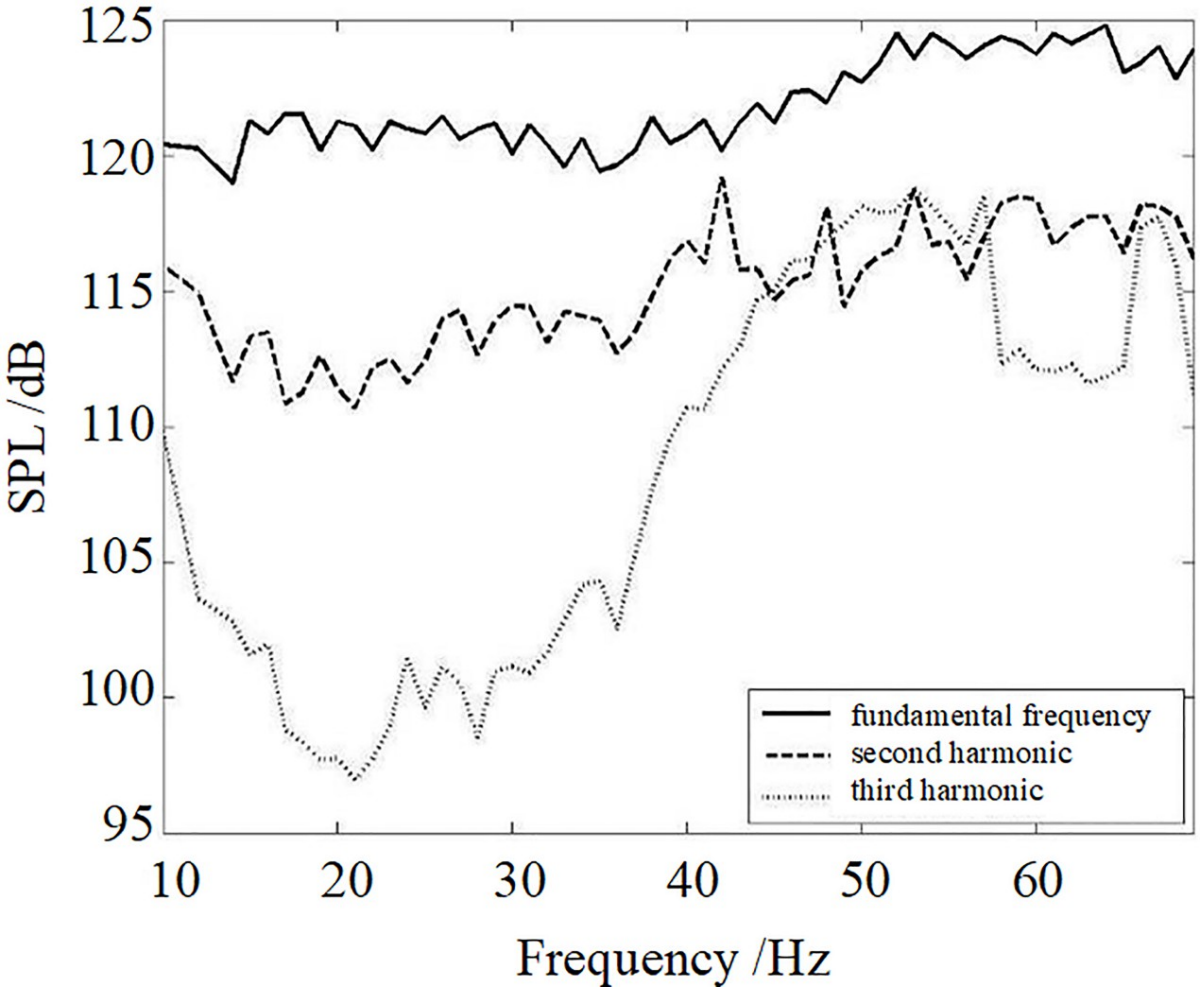
The SPL of the resonant acoustic generator at 1 m (45 degrees direction).

Fig 10 shows the sweep measurement results of the SPL of the fundamental frequency and its second and third harmonic at 1m (45 degrees direction). The fundamental frequency’s acoustic intensities from 10 Hz to 40 Hz are nearly 120 dB. The intensities of the fundamental frequency’s acoustic from 50 Hz to 65 Hz, which are near the target resonant frequency of 52 Hz, are nearly 124 dB. The actual resonant frequency should be in the range of 50 Hz to 65 Hz. The resonance can enhance the radiation acoustic intensity, and the improved value is 4 dB in this experiment. It can be seen from these curves that the intensity of the fundamental frequency acoustic is 5~10 dB larger than its second harmonic. The difference between the second and third harmonic fluctuates with the increase of frequency, and the difference value from 12 Hz to 38 Hz is larger. The maximum difference value is 15dB. The difference value from 45 Hz to 58 Hz is almost equal, and from 58 Hz to 62 Hz is 5 dB. The difference between the fundamental frequency and the third harmonic has a larger value from 12 Hz to 38 Hz and a max value of 25 dB. The second and third harmonic curves are staggered distributions above 42 Hz.

#### The measurement results of the acoustic propagation characteristic

**Fig 11:**
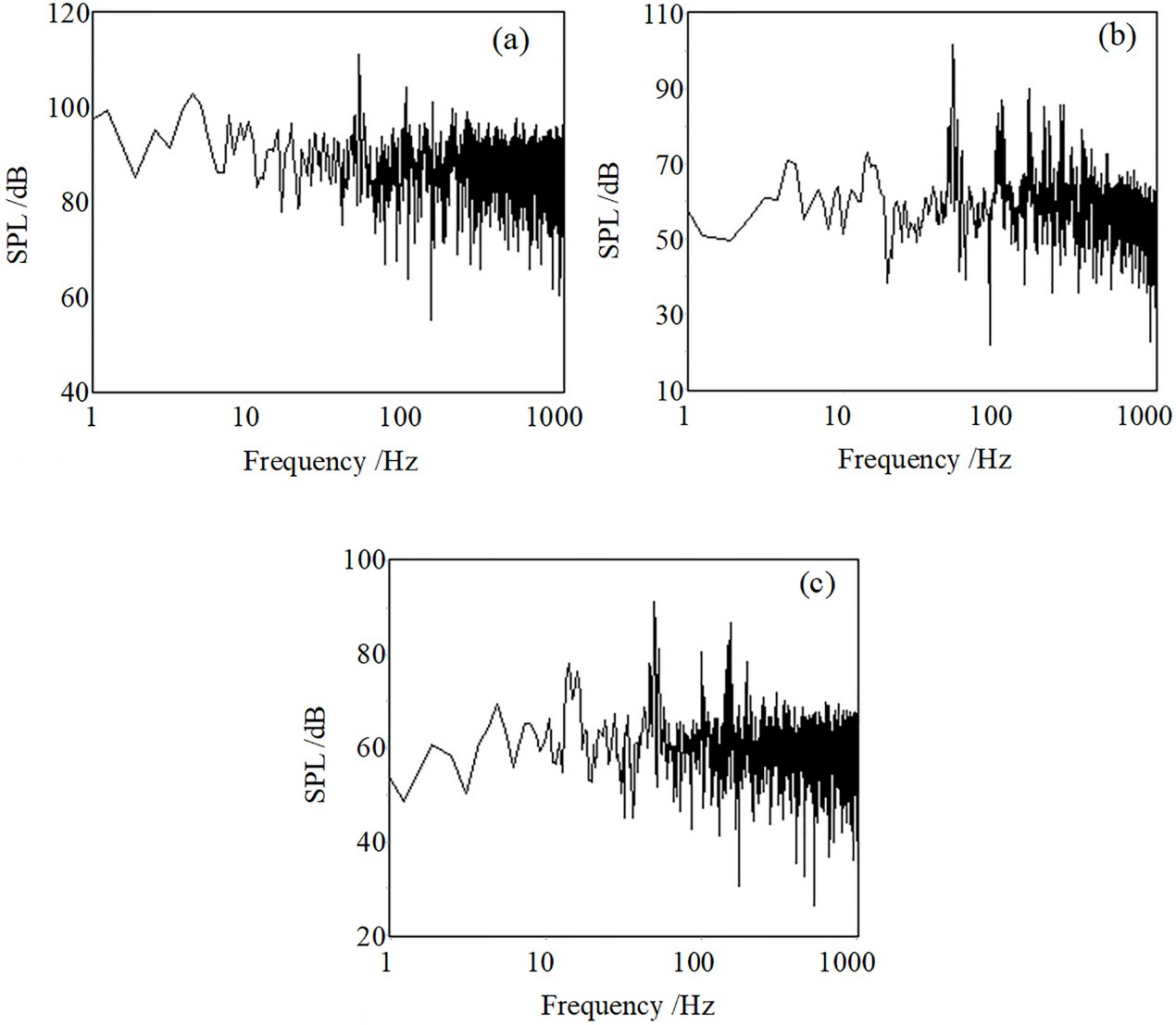
The spectrum is at 10 m (a), 50 m (b), and 100 m (c) (52 Hz).

Fig 11 shows the acoustic spectrum in the 10 m, 50 m, and 100 m distances. The intensities of the fundamental frequency 52 Hz and its second harmonic and third harmonic are 111 dB, 96 dB, and 92 dB at 10 m. The acoustic intensity of the fundamental frequency 52 Hz at 50 m and 100m is 102 dB and 91 dB. The acoustic intensity above 100 Hz at 10 m is almost 92 dB, eliminating 52 Hz and its harmonics. The intensity above 100 Hz at 50 m and 100 m is near 62 dB. So there are apparent wide spectrum jet noises at 10 m, which are very weak at 50m and 100m, and frequencies above 100 Hz are close to the environment background noise.

**Fig 12:**
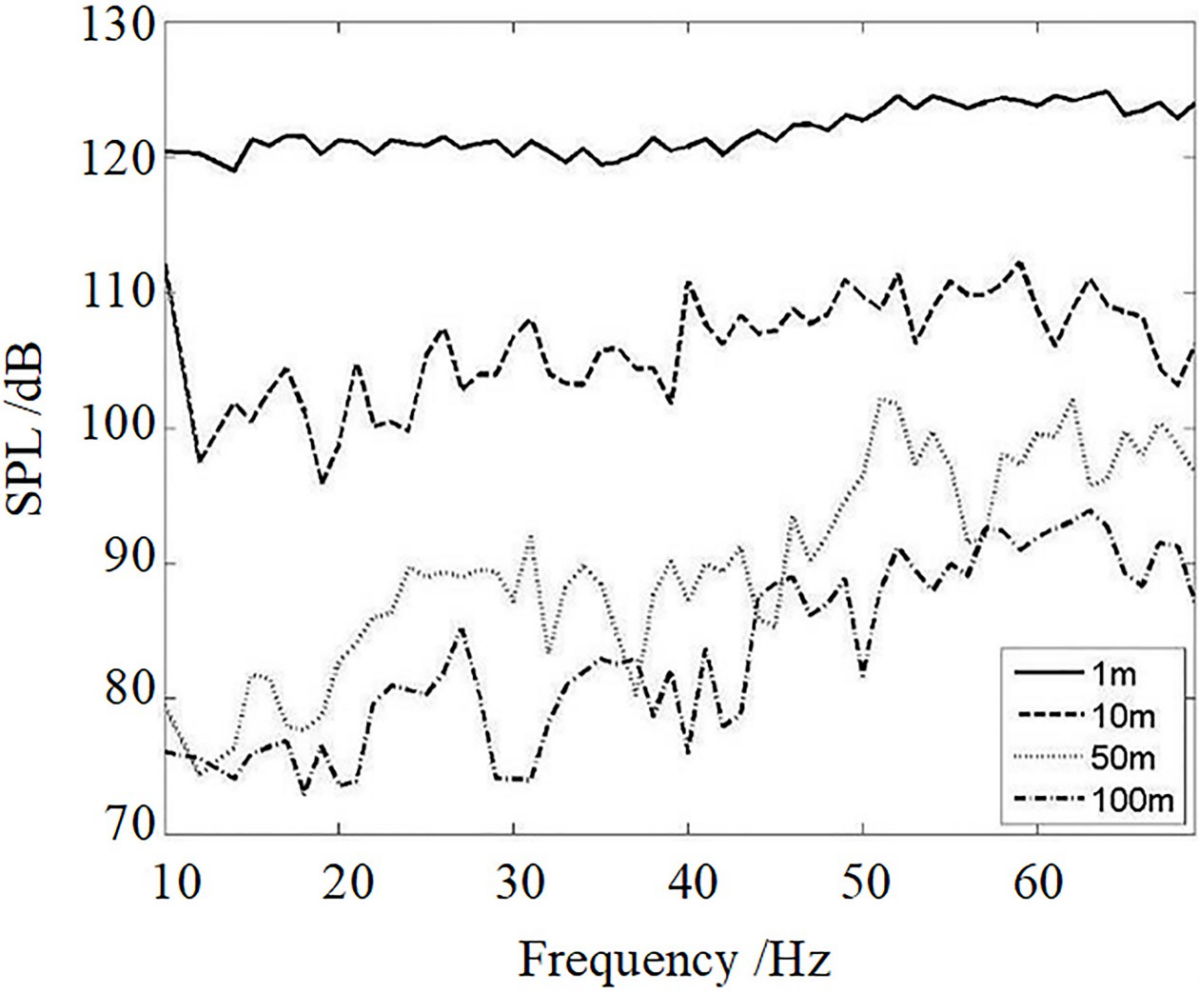
The fundamental frequency acoustic propagation characteristic.

Fig 12 shows the sweep measurement results of the SPL of the fundamental frequency at some measurement positions. The acoustic propagation characteristic of the resonant acoustic generator can be analyzed through this result. The difference value of the acoustic intensity between the resonant frequency and other frequencies is 4 dB when the frequencies are below 40 Hz. However, this difference value is enhanced with the increase in the distance. The maximum difference value is about 20 dB at 100 m.

Combined with the analysis of Figs 11 and 12, it can be seen that the acoustic generator in this study successfully generated obvious low-frequency sound waves in the open space within 100 m. The energy of the fundamental frequency, its second harmonic, and third harmonic of the low-frequency sound waves is significantly higher than the environment background noise.

## The analysis of the experimental results

Table 2 shows the three groups of measurement results at 10 Hz, 20 Hz, and 52 Hz. Although the SPL of the air chamber for the three conditions is decreased with the frequency increasing and the air chamber pressure of 10 Hz is 6 dB larger than 52 Hz, the max SPL of the nozzle of the resonant tube is 52 Hz’s SPL. The difference value between the SPL of 10 Hz and 20 Hz is only 1 dB, but the SPL of 52 Hz is 4 dB larger than 10 Hz. The max SPL at 1m, 50 m, and 100 m is 52 Hz. These results show that this device working at 52 Hz, has high radiation efficiency and intensity. The acoustic can propagate long distances. So the acoustic intensity of the resonant acoustic generator near the resonant frequency can be enhanced by the resonance.

**Table 2:** The experimental results for the characteristic of the resonant acoustic generator.

Content			
Working frequency /Hz	10	20	52
The SPL of the air chamber /dB	179	178	173
The SPL of the resonant tube /dB	164	165	168
SPL(1m) /dB	120	121	125
SPL(50m) /dB	79	84	102
SPL(100m) /dB	76	74	91

The internal pressure pulsation of the resonant tube can be enhanced by the air resonance of the resonant system. Then the flow disturbance degree of the radiated acoustic can be enhanced. So the acoustic radiation intensity and efficiency can be improved. It is the main reason why the resonant acoustic generator can enhance the intensity of the acoustic radiation.

## Conclusions

A big flow airflow modulator for turning the round throat was designed, and the characteristics of the throat were analyzed. Both the Helmholtz resonant cavity and the airflow modulator were combined. A periodic airflow generated by the modulator will generate acoustic waves and radiate through the resonant tube after the compressed air is rejected into the resonant cavity. The airflow resonance in the resonant cavity can enhance the air chamber’s interior air pressure pulsation. Thereby the air disturbance degree of the acoustic radiation is increased. So the acoustic radiation intensity and radiation efficiency can be lifted. To radiate a 52 Hz acoustic wave, an aero-acoustic generator was obtained. Some experiments were carried out to measure the internal flow field and radiated acoustic field characteristics for this generator. It is shown from the experimental results that the acoustic intensity for the 52 Hz at 1m position was 124 dB. The resonant enhancement effect was presented near the predetermined resonance frequency, and the enhanced value was about 4 dB.
